# Molecular mechanism of the camptothecin resistance of Glu710Gly topoisomerase IB mutant analyzed *in vitro* and *in silico*

**DOI:** 10.1186/1476-4598-12-100

**Published:** 2013-09-03

**Authors:** Cinzia Tesauro, Blasco Morozzo della Rocca, Alessio Ottaviani, Andrea Coletta, Laura Zuccaro, Barbara Arnò, Ilda D'Annessa, Paola Fiorani, Alessandro Desideri

**Affiliations:** 1 Department of Biology and Interuniversity Consortium, National Institute Biostructures and Biosystems (INBB), University of Rome Tor Vergata, Via Della Ricerca Scientifica, Rome 00133 Italy; 2 Institute of Translational Pharmacology, National Research Council, CNR, Via Del Fosso del Cavaliere 100, Rome 00133 Italy

**Keywords:** Religation rate, Molecular dynamics, Camptothecin, Drug resistance, Topoisomerase, Irinotecan, Mutation, Long-range correlation

## Abstract

**Background:**

DNA topoisomerases are key enzymes that modulate the topological state of DNA through the breaking and rejoining of DNA strands. Human topoisomerase IB can be inhibited by several compounds that act through different mechanisms, including clinically used drugs, such as the derivatives of the natural compound camptothecin that reversibly bind the covalent topoisomerase-DNA complex, slowing down the religation of the cleaved DNA strand, thus inducing cell death. Three enzyme mutations, which confer resistance to irinotecan in an adenocarcinoma cell line, were recently identified but the molecular mechanism of resistance was unclear.

**Methods:**

The three resistant mutants have been investigated in *S. cerevisiae* model system following their viability in presence of increasing amounts of camptothecin. A systematical analysis of the different catalytic steps has been made for one of these mutants (Glu710Gly) and has been correlated with its structural-dynamical properties studied by classical molecular dynamics simulation.

**Results:**

The three mutants display a different degree of camptothecin resistance in a yeast cell viability assay. Characterization of the different steps of the catalytic cycle of the Glu710Gly mutant indicated that its resistance is related to a high religation rate that is hardly affected by the presence of the drug. Analysis of the dynamic properties through simulation indicate that the mutant displays a much lower degree of correlation in the motion between the different protein domains and that the linker almost completely loses its correlation with the C-terminal domain, containing the active site tyrosine.

**Conclusions:**

These results indicate that a fully functional linker is required to confer camptothecin sensitivity to topoisomerase I since the destabilization of its structural-dynamical properties is correlated to an increase of religation rate and drug resistance.

## Background

The integrity and the physical organization of DNA must be maintained to ensure the survival of cells. Many essential cellular processes can cause problems in the topological structure of DNA. Separation of the two strands of the double helix generates tensions and other topological stresses that must be resolved in order to complete DNA metabolism processes such as replication, transcription and recombination [1,2]. These problems are solved by a class of ubiquitous enzymes called DNA topoisomerases. There are two classes of topoisomerases (type I and type II), both characterized by a catalytic mechanism which involves a nucleophilic attack of a DNA phosphodiester bond by a tyrosyl residue, but type I cleaves one DNA strand, whereas type II cleaves both strands [2]. Human DNA topoisomerase IB (hTop1) is composed of 765 amino acids, and the crystal structure of the N-terminal truncated protein (Topo70), together with proteolytic experiments, has shown that the enzyme is composed of four different domains: the N-terminal domain (residues 1–214), the core domain (215–635), the linker domain (636–712), and the C-terminal domain (713–765) [3-5]. Mechanistically, hTop1 catalyzes DNA relaxation by transiently cleaving, passing, and religating one strand of the DNA double helix. The active site tyrosine (Tyr723) starts the catalytic cycle of the enzyme through a nucleophilic attack on the DNA backbone, resulting in the breakage of one DNA strand with the enzyme covalently attached to the 3′-phosphate to form the cleavage complex. After changing the linking number, a second nucleophilic attack, driven by the 5′-hydroxy DNA end, restores an intact double-stranded DNA, and the enzyme is released [5].

Formation of the cleavage complex is a critical event during the cell cycle since cell vitality is seriously compromised by poisoning this complex [6]. Many small natural and synthetic molecules target DNA topoisomerases [7,8]. Among these agents, camptothecin (CPT), the most important poison of hTop1, represents the lead compound of an important class of antitumor drugs. In physiological conditions the DNA nicks produced by hTop1 are rapidly sealed, while CPT can reversibly bind to the covalent hTop1-DNA complex slowing down the religation of the cleaved DNA strand. The stalled hTop1 complex can collide directly with the progression of the replication fork producing lethal double strand DNA breaks and ultimately cell death [9]. Recently, single molecule data have hinted to a more indirect mechanism in which the tension due to positive supercoils, generated ahead of the fork and less efficiently removed by the ternary complex, mediate the fork collapse and formation of DNA lesion [10]. Either way, CPT converts hTop1 to a cell poison and consequently the citotoxicity of CPT correlates directly with the intracellular hTop1 activity level. Two water-soluble CPT derivatives, topotecan (TPT) and irinotecan (CPT-11), have been approved by the FDA and widely used for cancer chemotherapy. TPT is used for the treatment of cisplatin-refractory ovarian carcinoma, and for second-line therapy in small-cell lung cancer (SCLC). CPT-11, with is active metabolite SN38, has been approved in the USA for the treatment of colorectal cancer [11]. Although TPT and CPT-11 have shown effectiveness in the treatment of the above mentioned cancer types, drug resistance is still a critical problem [12]. The mechanism behind the clinical resistance to CPTs has not been fully elucidated since selectivity and resistance towards cancer cells are multifactorial [11-13]. Among all, the most studied determinants of drug resistance concern hTop1 mutations with consequent reduction of cleavage complexes [11,12].

An important contribution toward the understanding of the interaction of CPT with hTop1 and DNA has been provided by the crystal 3D structure of the ternary complex made by Topo70, corresponding to the enzyme depleted of the N-terminal domain, covalently linked to DNA and TPT [14,15]. The structure reveals that the drug molecule intercalates between upstream (−1) and downstream (+1) bp, moving the 5′-hydroxyl end away from the scissile phosphate and thus preventing the religation of the cleaved strand. This structure permits to explain the CPT resistance due to mutations involving residues that interact directly with the drug or that alter the interaction with DNA [16]. However, it is not able to explain the CPT resistance for point mutations involving residues not directly contacting the drug. In this case an explanation has been provided by a series of works combining *in vitro* activity assays and molecular dynamics (MD) simulation [16-19]. These works have shown that a common feature of these CPT resistant mutants is the presence of an anomalous linker mobility and/or a loss of interdomains correlated motion between the linker domain and the C-terminal domain containing the catalytic tyrosine 723. The importance of the linker in modulating the CPT sensitivity has been firstly demonstrated by its deletion that gives rise to an enzyme that has an increased religation rate and is partially CPT resistant [20]. The linker-deleted enzyme also loses the correlation between the various protein domains as demonstrated by MD simulations [21]. A connection between the presence of the drug and linker mobility has been confirmed by the simulation of the hTop1-DNA-TPT ternary complex showing that the presence of the drug reduces the linker mobility [22] a result confirmed by the simulation of the hTop1-DNA-indenoisoquinoline ternary complex [23]. In line the 3D structure of the ternary complex shows a well defined electron density for the linker domain that is not observed for the DNA-topo70 binary complex crystallized in the same conditions [4,5].

In a recent report new hTop1 mutations which confer resistance to irinotecan in a adenocarcinoma cell line have been identified [24]. These mutations are located in helix 17 of the core subdomain III (Arg621His and Leu617Ile) and at the end of helix 19 of the linker domain (Glu710Gly). The arginine 621 and the glutammic acid 710 interact together via a salt bridge and form part of the interface between helices 17 and 19. Arg621His and Glu710Gly are found in moderately resistant clones, whereas Leu617Ile mutation is found in highly resistant clones that also over-express the ABCG2 transporter, so that in this latter case the resistance is also due to the elimination of the drug by the efflux pumps [24]. The authors suggest that the resistance is likely due to an altered hTop1 linker flexibility but don’t provide any clear evidence for such hypothesis.

In the present paper we have produced Arg621His, Glu710Gly and Leu617Ile mutants and confirmed their CPT sensitivity in a yeast cell viability assay. Due to our experience and interest in the characterization of drug resistant mutants involving residues localized in the linker domain, we then focused our research on the purified Glu710Gly mutant in order to find a molecular explanation for its resistance. Through a combined experimental and simulative approach, we provide evidence that the CPT resistance of the mutant is due to a fast religation rate coupled to a loss of correlation between the linker and the C-terminal domain confirming the crucial role of the linker domain in controlling hTop1 drug sensitivity.

## Results

### Glu710Gly mutant is resistant to CPT *in vivo* and *in vitro*

**Figure 1:**
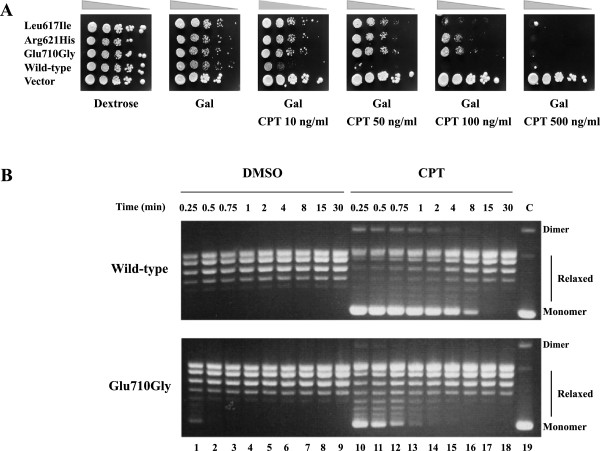
**CPT sensitivity *in vivo *and *in vitro*. (A)** Exponentially growing yeast cells transformed with a single copy plasmid expressing Leu617Ile, Arg621His, Glu710Gly, wild type and vector have been serially 10-fold diluted and spotted onto selective media in the presence of dextrose, of galactose (Gal), or of galactose plus the indicated CPT concentrations. **(B)** Relaxation of negative supercoiled plasmid in a time course experiment for the wild type and the Glu710Gly mutant in presence of DMSO (lanes 1–9) and 100μM CPT (lanes 10–18); lane 19 represents the control **(C)** where no protein added. The reaction products are resolved in an agarose gel and visualized with ethidium bromide. The two forms of the supercoiled plasmid DNA are indicated as “Dimer” and “Monomer”.

The mutations (Leu617Ile, Arg621His and Glu710Gly) found in the CPT-11 resistant HCT116 clones by Gongora *et al.*[24] have been introduced in the single copy yeast plasmid (YCp) expressing hTopI under the GAL1 promoter. To assess the *in vivo* consequences of mutants expression, the viability and CPT sensitivity of Top1Δ yeast cells (EKY3), transformed with GAL1-hTop1 constructs, have been tested. The expression vector carries the Ura-selectable marker and is maintained by selection in synthetic complete SC Ura-medium. At least five independent clones were selected from each transformation. Serial dilutions of yeast cells, transformed with the indicated plasmids, have been spotted on plates containing dextrose or galactose, supplemented with different CPT concentrations, to assess drug sensitivity following the yeast growth. The data show that yeast cells expressing the wild type protein exhibit a deficiency in viability in the presence of 10 ng/ml CPT, while the three mutations render the enzyme resistant to CPT (Figure 1A). However, the mutants display a different sensitivity to CPT concentration: Arg621His and Glu710Gly produce viable colonies until 100 ng/ml of CPT while the Leu617Ile is less resistant growing only up to 50 ng/ml.

The effect of 60 μM CPT on the ability of wild type and Glu710Gly enzymes to mediate relaxation of supercoiled plasmid, under standard assay conditions, has been assessed incubating 18 ng of each enzyme with 0.5 μg of a negative supercoiled plasmid in a time course kinetics from 0.25 to 30 minutes. Since CPT is dissolved in dimethyl sulfoxide (DMSO), an assay of the enzyme activity in the presence of an identical amount of DMSO without CPT has been also carried out, to show that DMSO does not affect the relaxation activity of the enzyme. The reactions have been stopped and the products resolved by agarose gel electrophoresis. As shown in Figure 1B, the two enzymes exhibit comparable relaxation activities, since they completely relax the supercoiled DNA after 0.5 minutes of incubation (Figure 1B, lanes 2). The presence of CPT considerably slows down the relaxation of native hTop1, a full relaxation being observed only after about 8 minutes from the drug addiction (Figure 1B, lane17). On the other hand the Glu710Gly mutant completely relaxes the supercoiled DNA in 1–2 min in presence of the drug (Figure 1B lane 13–14), confirming its CPT resistance. The same experiment has been carried out using a more diluted enzyme, confirming that the relaxation rate for the wild type and mutant is comparable and that the mutant is CPT resistant (data not shown). The resistance of the Glu710Gly has been analyzed in detail following the different steps of the catalytic cycle and studying its structural and dynamical properties by MD simulation.

### Kinetics of cleavage of the wild type and Glu710Gly mutant

**Figure 2:**
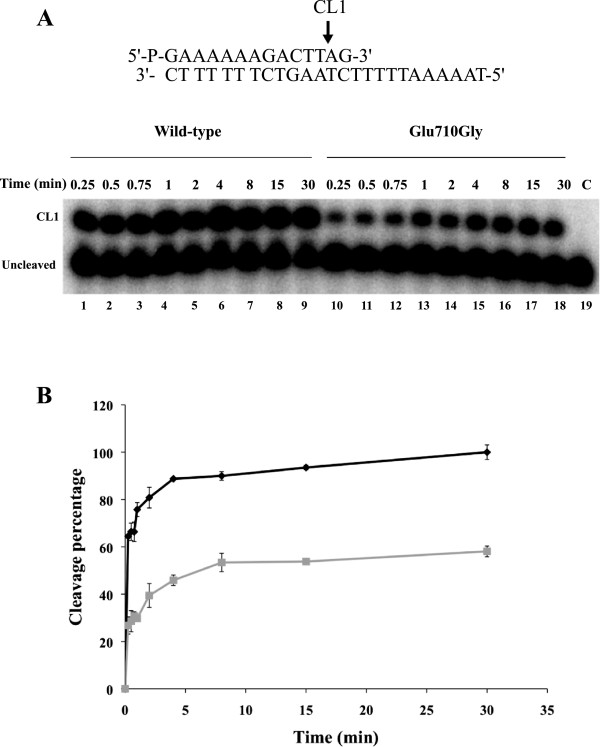
**Cleavage kinetics. (A)** Time course (0.25-30 minutes) of the cleavage reaction between the CL14/CP25 suicide substrate, shown at the top of the figure and the purified wild type (lanes 1–9), and Glu710Gly mutant (lanes 10–18). In lane 19 the protein has not been added. CL1 represents the DNA strand cleaved by the enzymes at the preferred cleavage site, indicated by an arrow. **(B)** Percentage of cleaved suicide substrate, normalized to the maximum value of the wild type, plotted against time for the reaction with wild type (black) and Glu710Gly mutant (grey). Data shown are means ± SD from 3 independent experiments.

**Figure 3:**
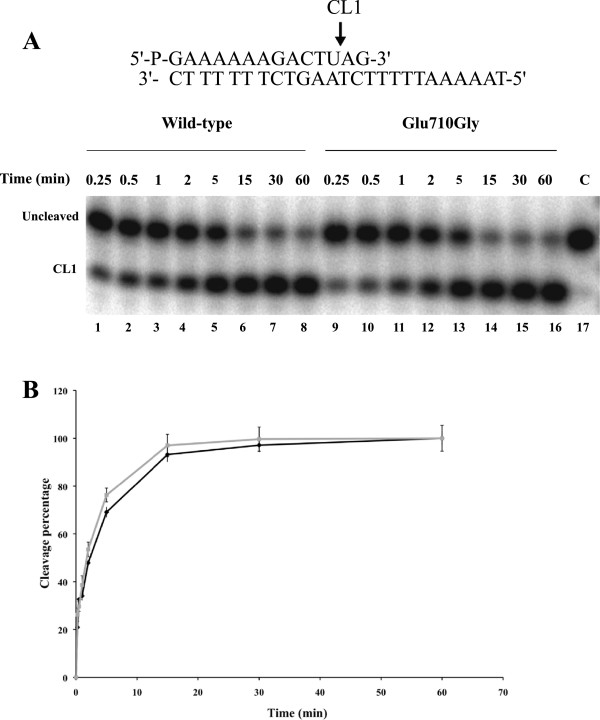
**Cleavage kinetics using ribo-modified substrate. (A)** Time course (0.25-60 minutes) of the cleavage reaction of purified wild type (lanes 1–8), and Glu710Gly mutant (lanes 9–16) with the CL14-U/CP25 substrate (shown at the top of the figure). In lane 17 the protein has not been added. CL1 represents the DNA strand cleaved by the enzymes at the preferred cleavage site, indicated by an arrow at the top of the figure. **(B)** Percentage of cleaved substrate, normalized to the maximum value of the wild type, plotted against time for the reaction with wild type (black diamond’s) and with Glu710Gly mutant (grey squares). Data shown are means ± SD from 3 independent experiments.

The time course of the cleavage of the wild type and Glu710Gly mutant has been followed using a suicide cleavage substrate. In detail, a 5′-end radiolabeled oligonucleotide CL14 (5′-GAAAAAAGACTTAG-3′) has been annealed to the CP25 (5′-TAAAAATTTTTCTAAGTCTTTTTTC-3′) complementary strand, to produce a duplex with an 11-base 5′-single-strand extension. The enzyme preferentially cuts at the site indicated by the arrow, as shown in top of Figure 2A. Using this substrate the religation step is almost totally precluded, because the AG-3′ is too short to be religated, leaving the enzyme covalently attached to the 12 oligonucleotide 3′-end [17]. For a suicide substrate incubated with an excess of wild type or mutant enzyme, the cleaved DNA fragments have been resolved in a time course experiment in a denaturing polyacrylamide gel as reported in Figure 2A. The amount of fragment, normalized to the maximum value of the wild type protein, plotted as a function of time in Figure 2B, suggests that both enzymes have an almost identical cleavage rate (k_cl_), being the initial part of the curves almost identical in both reactions. The two enzymes however don’t reach the same plateau value leaving us with the hypothesis that a small amount of AG-3′ could be religated and that the mutant could religate this short oligonucleotide faster than the wild type enzyme. In order to address this hypothesis a different cleavage substrate has been used to follow the rate of cleavage. In detail, a 5′-end radiolabeled oligonucleotide, named CL14-U (5′-GAAAAAAGACTUAG-3′), having the deoxyribo-thymine (dT) in position 12 substituted with a ribo-Uracil (rU), has been annealed to the CP25 (5′-TAAAAATTTTTCTAAGTCTTTTTTC-3′) complementary strand, to produce a duplex with an 11-base 5′-single-strand extension. With this substrate, when the enzyme cuts at the preferential site indicated by an arrow at the top of Figure 3A, the 2′-OH of the ribose attacks the 3′-phosphotyrosyl linkage between the enzyme and ribonucleotide, releasing hTop1 and leaving a 2′,3′-cyclic phosphate end [25]. In this way the enzyme is not anymore covalently linked to the substrate and the short oligonucleotide cannot be religated. The wild type and the mutant have been incubated with the substrate and the fragments have been resolved in a time course experiment in a denaturing polyacrylamide gel and the result is reported in Figure 3A. The cleaved oligo (CL1 in Figure 3A) runs like a 12 mer since no protein is attached to it and the plot of the amount of this fragment as a function of time, normalized to the maximum value of the wild type protein, unambiguously demonstrates that the wild type protein and the Glu710Gly mutant have the same rate of cleavage and reach the same plateau value (Figure 3B).

### Kinetics of religation of the wild type and Glu710Gly mutant

**Figure 4:**
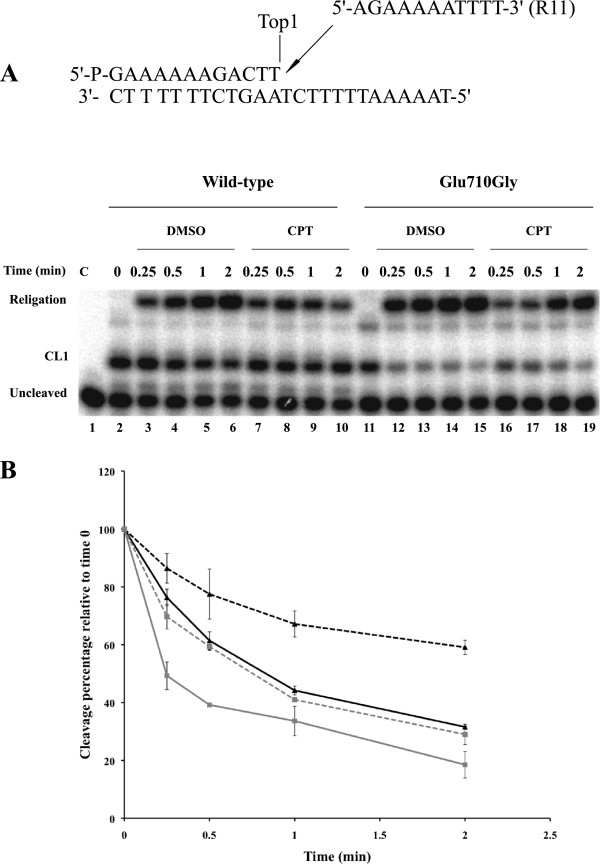
**Religation kinetics. (A)** Gel analysis of the religation kinetics observed when incubating the wild type or the Glu710Gly mutant-suicide cleavage complex with the R11 complementary ligator oligonucleotide (shown at the top of the figure) in absence [lanes 3–6 and lanes 12–15 for wild type and Glu710Gly mutant respectively] or in presence of 100 μM CPT [lanes 7–10 for the wild type and lanes 16–19 for the Glu710Gly mutant]. In lane 1 no protein was added. The lanes 2 and 11 represent the time 0 for the wild type and the Glu710Gly mutant reactions, before the addition of the complementary R11 strand. “CL1” represents the DNA fragment cleaved at the preferred enzyme site; “religation” is the restored fully duplex oligonucleotide representing the final product of the religation reaction. **(B)** Plot of the percentage of disappearance of the cleavage complex relative to time 0, in absence or in presence of CPT for the wild type (triangles, full and dashed black lines, respectively) and the Glu710Gly mutant (squares full and dashed grey lines, respectively). Data shown are means ± SD from 3 independent experiments.

The DNA religation step has been studied by testing the ability of both enzymes to religate the oligonucleotide R11 (5′-AGAAAAATTTT-3′) in the presence or absence of CPT, added to the cleavage complex obtained incubating the suicide substrate with an excess of enzyme. Aliquots have been removed at different times, the reaction stopped by addition of SDS and the products analyzed by polyacrylamide gel electrophoresis (Figure 4A). The percentage of the remaining covalent complex (CL1), normalized to the value at t=0 is plotted, as a function of time, in Figure 4B. The data show that the Glu710Gly mutant has a higher religation rate when compared with the wild type enzyme (Figure 4A, compare lanes 2–6 with lanes 11–15). The presence of CPT strongly decreases the religation rate in the wild type protein (lanes 7–10), whereas has only a small effect for the Glu710Gly mutant (lanes 16–19) bringing the religation rate to a value similar to the one observed for the wild type in absence of the drug, as shown by the quantitative plot of Figure 4B.

### MD simulation

**Figure 5:**
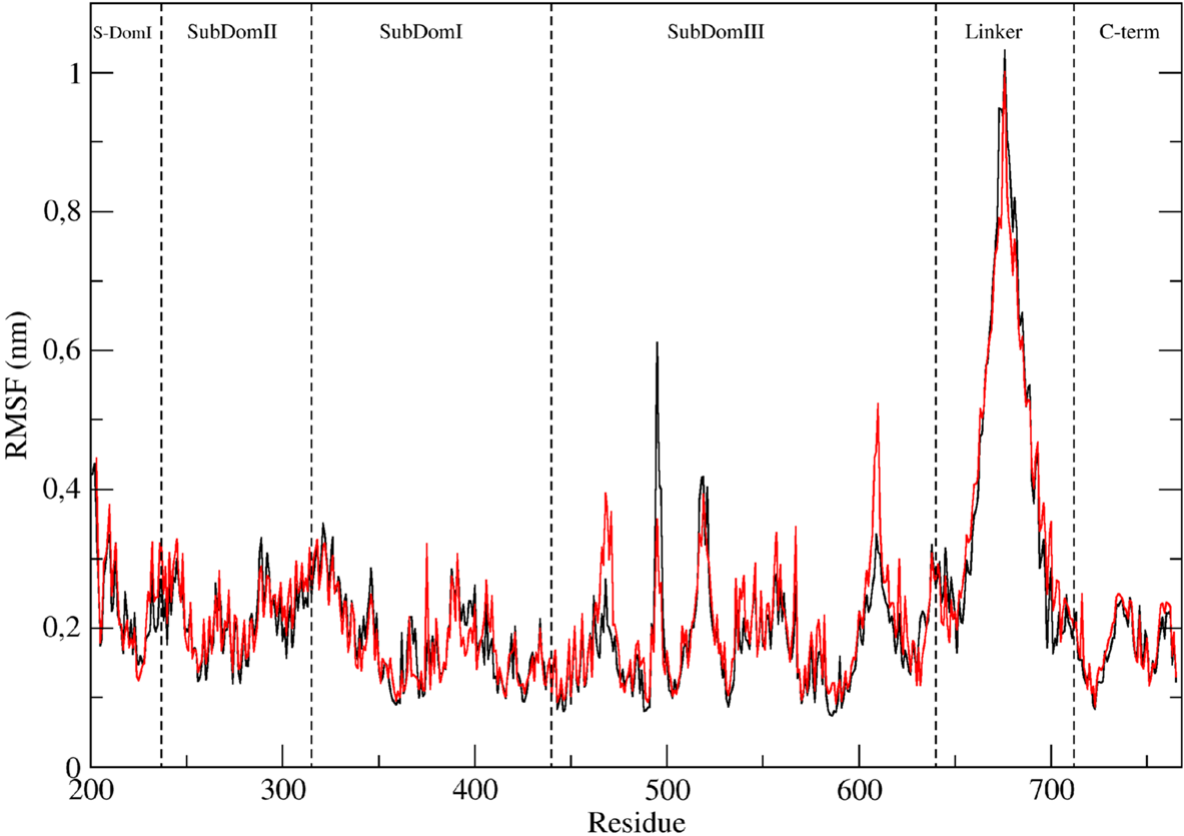
**Root mean square fluctuation of the protein residues during the MD trajectory.** Black line represents the wild type and the red one the mutant. The various domains of the protein are indicated for sake of clarity.

Root mean square displacement (RMSD) plots along the 75 ns simulation of the native and mutated proteins show that in both proteins the core is stable (see Additional file 1) and that the largest deviation is due to the linker domain. This is confirmed by the root mean square fluctuations (RMSF), where the larger contribution comes from the linker domain (Figure 5). The two systems show comparable values along the sequence, except for the peaks at 466–471 and 608–610 that have higher values for the mutant and the 494–497 that have higher values in the wild type.

### Local impact of mutation

**Figure 6:**
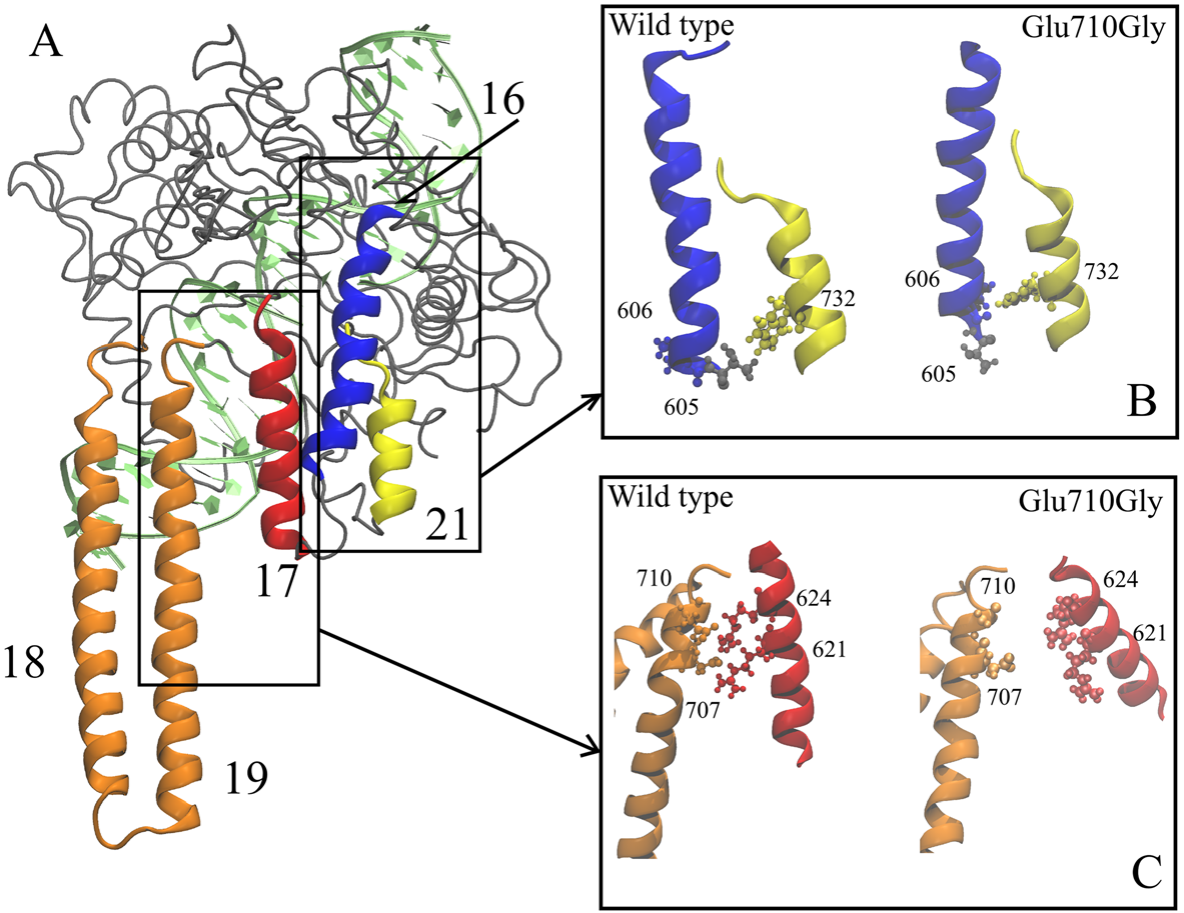
**Protein structure and representative interactions that change during the simulations. (A)** View of the protein depicted as a gray tube, DNA strand in green and helices of interest are depicted as ribbons: helices 18 and 19 (linker domain) in orange, helix 16 in blue, helix 17 in red, helix 21 in yellow. **(B)** A closeup of the representative snapshots of the simulations depicting the interactions between helix 16 and helix 21. **(C)** Same as B but closeup on the interactions between helix 17 and helix 19.

In the X-ray structures [5,15,26] helix 19 (residues 679–710, one of the two helices of the linker domain), directly faces helix 17 (residues 612–629), through two salt bridges (Glu710-Arg621 and Asp707-Arg624) permitting the communication between the linker domain and the cap-core structure of the protein (Figure 6A). In the wild type simulation the side chains of these residues keep forming a network of salt bridges for a large percentage of time (85% and 96%) as shown in Figure 6C, left side, where a representative snapshot of the simulation is shown. In the simulation of the Glu710Gly mutant, besides the obvious disappearance of the Glu710-Arg621 salt bridge, the Asp707-Arg624 salt bridge is also broken (only 9% existence), thus separating the communication between the base of the linker with the core of the protein (Figure 6C, right side). Another effect of the mutation is observed in the bundle of helices (16, 17 and 21) neighbouring the mutation zone, (see Figure 6A), where helices 16 and 21 change their relative orientation since Trp732, located on helix 21, interacts with Thr605 in the wild type, but with Leu606 in the mutant simulation as shown by two representative snapshots of the simulations in Figure 6B. As a consequence the C-terminal of helix 16, downstream of this interaction increases its flexibility explaining the peak seen in the RMSF for residues 607–610 (Figure 5).

### Global motion

**Figure 7:**
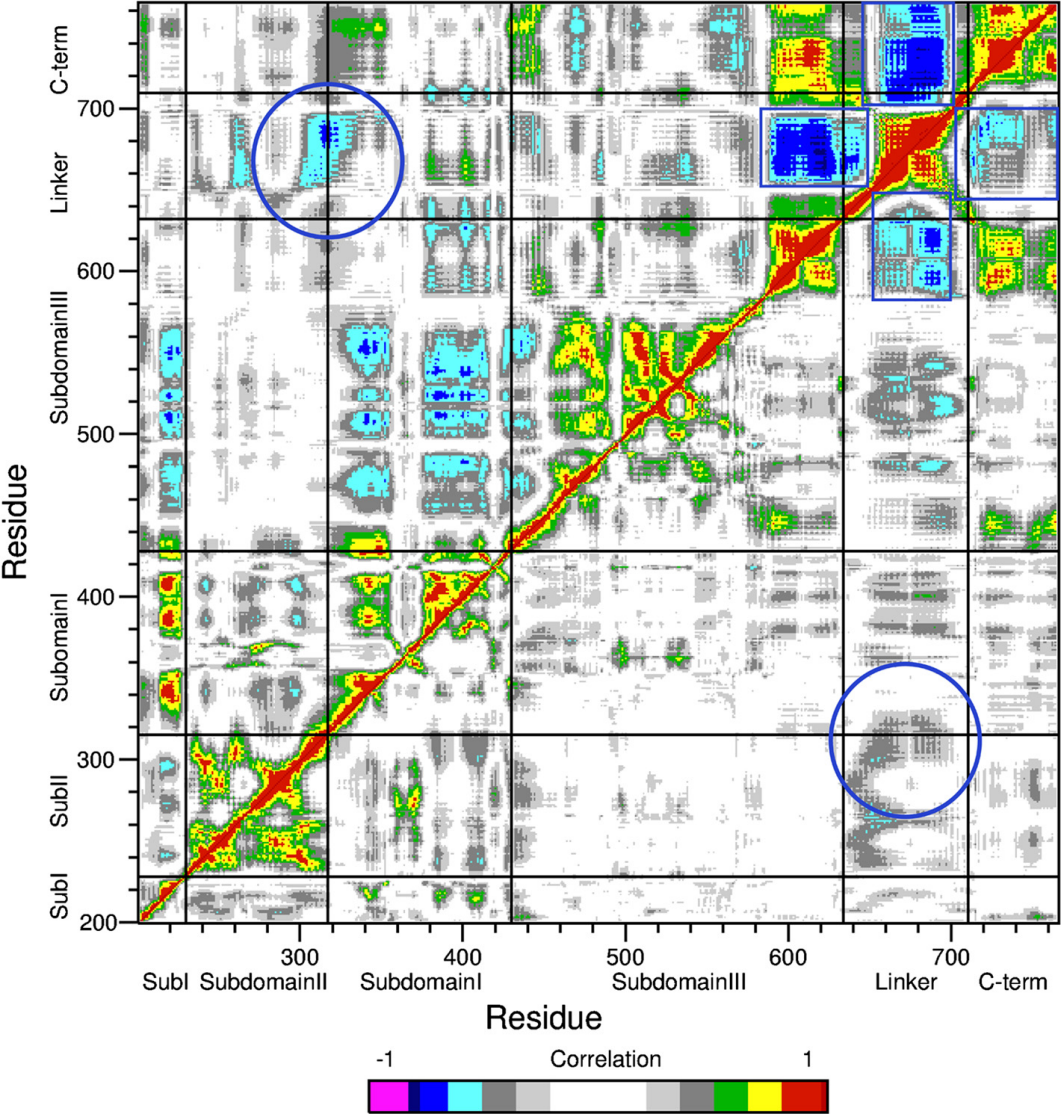
**Dynamic cross correlation maps between Cα atoms, of the wild type and mutated protein.** Top left triangle represents the wild type, bottom right represents the Glu710Gly mutant. Subdomain decomposition is also indicated. Color coding for the correlation values is given on the bottom, negative values indicate anticorrelation, positive ones indicate direct correlation. Regions of interest are enclosed in blue rectangles and circles.

The Glu710Gly mutation has an important effect on the concerted motions of the protein as can be noted comparing the dynamic cross-correlation matrix of the C-alpha atoms of the protein residues during the trajectories of the wild type and the mutant (Figure 7, top and bottom halves respectively). In the figure the pairs of Cα atoms characterized by strong correlated motions are represented by a pixel of red, yellow or green color, while anticorrelated motions are shown in cyan, blue and violet. The mutant is endowed with a much lower degree of correlation. In detail, in the wild type, the linker has a strong anticorrelated motion with the C-teminal domain and the C-terminal region of subdomain III (squares in Figure 7) and a lower, but still pronounced, anticorrelated motion with the nose cone helices (representing the protein region that contacts the DNA on the opposite side respect to the linker, downstream of the cleavage site, circled in Figure 7). In the mutant these anticorrelated motions are lost or strongly weakened (Figure 7 bottom). A similar loss of correlation has been found for the Thr729Lys that also displays CPT resistance [18].

## Discussion

In this study we have characterized three hTop1 mutants, Leu617Ile, Arg621Lys and Glu710Gly, found to be involved in the cytotoxicity mechanisms of CPT-11 in colon cancer cell lines [24]. A viability assay carried out on yeast cells, where the endogenous Top1 has been deleted and the hTop1 with its specific mutations has been inserted, indicates that the cells are able to grow also in presence of CPT (Figure 1A), confirming the CPT resistance of the three mutants. Arg621Lys and Glu710Gly display a CPT resistance higher than Leu617Ile mutant (Figure 1A) while in the results observed in colon cancer cells Arg621Lys and Glu710Gly are found in the moderately resistant clones, and Leu617Ile mutant in highly resistant clones [24]. These apparent contradictory results can be explained considering that this latter clone is also overexpressing the ABCG2 pump, so that part of the resistance is due to the expulsion of the drug by the pump, whilst for the other two clones the resistance is only due to the hTop1 mutations.

Characterization of the different steps of the catalytic cycle of the Glu710Gly mutant permits to understand the basis for its resistance. The mutant has a relaxation rate comparable to the wild type but addition of CPT only partially affects its relaxation at variance on what observed on the wild type (Figure 1B lane 10–18). The mutant and the wild type have a comparable cleavage rate. However when a suicide substrate is used they reach different plateau values, being lower for the mutant (Figure 2). This result can be interpreted considering that the mutant has a cleavage rate identical to the wild type but it is characterized by a faster religation rate, that allows the mutant to rapidly religate the dinucleotide lowering its plateau. In line with this hypothesis using a different cleavage substrate having a ribonucleotide in the −1 position, wild type and mutant not only have the same rate but also reach an identical plateau (Figure 3). With this substrate the detachment of the protein from the cleavage complex is not carried out by the 5′-OH of the dinucleotide but by the 2′-OH of the ribose moiety in position −1, and this detachment is likely happening with the same efficiency for the two enzymes [25].

The large rate of the mutant in religation is demonstrated by the experiment in Figure 4 where it is shown that the complementary strand added to the cleavage complex is bound by the mutant at least twice faster than the wild type. Moreover addition of CPT only slightly reduces the mutant religation rate, that in presence of the drug reaches a value comparable to the one observed for the wild type in absence of CPT (Figure 4).

The increase in religation rate is correlated to a change in the structural-dynamical properties of the enzyme as studied by MD simulation. The mutant is in fact characterized by a much lower degree of correlation of the protein domain motions when compared to the wild type. In detail, the large correlation observed in the wild type between the linker domain and the C-terminal domain, the C-terminal region of subdomain III and the nose cone helices is almost completely lost in the mutant (Figure 7). This result is likely due to the breaking of a direct communication between the linker domain and the core of the protein due to a loss of two salt bridges (Figure 6). Following these data the linker and the active site, located in the C-terminal domain, must have an anticorrelated motion to have a full control of the religation rate. It is interesting that a similar behavior has been observed for another single CPT resistant mutant where Thr729 has been mutated to Lys [27]. Also in this case a disabled linker not having an anticorrelated motion with the C-terminal domain and the C-teminal region of the core domain produced CPT resistance and an increased religation rate, confirming the important role of the linker domain in tuning the religation process.

The importance of the linker in modulating the enzyme function has been firstly demonstrated studying the enzyme deleted of the linker that displays an increase of the religation rate and a decrease in CPT sensitivity [20]. In a series of works concerning single and double mutants (e.g. Ala653Pro, Lys681Ala, Asp677Gly-Val703Ile) we have shown that there is a correlation between linker flexibility and CPT reactivity [16,17,28] a large flexibility being associated to CPT resistance. Such a correlation has been recently confirmed from a chimeric enzyme constituted by the human enzyme containing a linker from *P. falciparum* Top1 [29]. The chimeric enzyme has a linker displaying a large flexibility and it is CPT resistant. In this work we show that not only an increased flexibility but also a loss of the linker correlated motions produce an increased religation rate and CPT resistance, as also found for the Thr729Lys mutant [27]. CPT has been proposed not only to interfere with religation but also to slow down the uncoiling rate [10]. This effect is seen only in presence of a fully functional linker and so the loss of linker correlated motions observed in the here studied mutant could be related to CPT resistance trough its incapability in slowing down the uncoiling rate as observed in single molecule experiments [10,30].

Taken together all these works point out the crucial role of the linker in modulating and controlling the enzyme catalysis and in particular the religation step and confirm that, any time we have a disabled linker, either through a large flexibility or via a loss of correlated motions with other domains and in particular with the C-terminal domain containing the active site, a direct effect on the catalytic rate and then on the CPT reactivity is found.

## Materials and methods

### Chemicals, yeast strains and plasmids

DMSO and CPT were purchased from Sigma-Aldrich. CPT was dissolved in 99.9% DMSO to a final concentration of 4 mg/ml (11.5 mM) and stored at −20°C.

ANTI-FLAG M2 monoclonal affinity gel, FLAG peptide and ANTI-FLAG M2 monoclonal antibody were purchased from Sigma-Aldrich.

*Saccharomyces cerevisiae* top1 null strain EKY3 (*ura3-52, his3Δ200, leu2Δ1, trp1Δ63, top1:TRP1, MATα*) previously described [31] was used to express the hTop1 gene. YCpGAL1-e-hTop1 single copy plasmid was described previously [31,32]. Leu617Ile, Arg621His and Glu710Gly were generated by oligonucleotide-directed mutagenesis of the YCpGAL1-wild type in which the human topoisomerase I is expressed under the galactose inducible promoter in a single-copy plasmid. The epitope-tagged construct YCpGAL1-e-wild type contains the N-terminal sequence FLAG: DYKDDDY (indicated with ‘e’), recognized by the M2 monoclonal antibody. The epitope-tag was subcloned into YCpGAL1-hTop1Leu617Ile, YCpGAL1-hTop1Arg621His and YCpGAL1-hTop1Glu710Gly to produce the YCpGAL1-e-hTop1Leu617Ile, YCpGAL1-e-hTop1Arg621His and YCpGAL1-e-hTop1Glu710Gly construct. The cloning reactions were transformed into XL10-Gold *E. coli* cells (Agilent Technologies) and positive clones were identified by sequencing the extracted plasmid DNA.

### Drug sensitivity assay

Yeast EKY3 strains were transformed with YCp50, YCpGAL1-e-hTop1, YCpGAL1-e-hTop1Leu617Ile, YCpGAL1-e-hTop1Arg621His and YCpGAL1-e-hTop1Glu710Gly vectors by LiOAc treatment [33] and selected on synthetic complete (SC)-uracil medium supplemented with 2% dextrose. Transformants were grown to an A^595^=0.3 and 5 μl aliquots of serial 10-fold dilutions were spotted onto SC-uracil plates plus 2% dextrose or 2% galactose, with or without the indicated concentrations of CPT.

### HTop1 and hTop1Glu710Gly purification

EKY3 yeast cells, transformed with the YCpGAL1-e-hTop1 and YCpGAL1-e-hTop1Glu710Gly were grown overnight on SC-uracil plus 2% dextrose, at an optical density of A^595^=1.0 they were diluted 1:100 in SC-uracil plus 2% raffinose. At an optical density of A^595^=1.0, the cells were induced with 2% galactose for 6 h. Cells were then centrifuged, washed with cold water and resuspended in 2 ml buffer/g cells (50 mM Tris–HCl, pH 7.4, 1 mM EDTA, 1 mM EGTA, 10% glycerol and protease inhibitors cocktail from Roche, supplemented with 0.1 mg/ml sodium bisulfate, 0.8 mg/ml sodium fluoride, 1mM Phenylmethanesulfonylfluoride (PMSF) and 1mM DTT). After addition of 0.5 volumes of 425–600 μm diameter glass beads, the cells were disrupted by vortexing for 30 seconds alternating with 30 seconds on ice and then were centrifuged at 15000g for 30 minutes. For homogenous protein preparations, the whole extracts were applied to an ANTI-FLAG M2 affinity gel (Sigma-Aldrich) already equilibrated in according with the manufacturer protocol. Then, columns were washed with 20 volumes of TBS (50 mM Tris–HCl pH 7.4 and 150 mM KCl) supplemented with the protease inhibitors, prior to load the lysate. Elution of e-hTop1, or e-hTop1Glu710Gly, was performed by competition with five column volumes of a solution containing 1mg of FLAG peptide (DTKDDDDK) in TBS. Fractions of 500 μl were collected and 40% glycerol was added in all preparations, which were stored at −20°C [17]. Protein levels and integrity were assessed by immunoblot with the monoclonal anti M2 antibody (Sigma-Aldrich). The hTop1 and hTop1Glu710Gly similar concentrated fractions were also compared to the purified hTop1 with a known concentration (provided from Topogene) by immunoblot using the ab58313 Anti-Top1 antibody (Abcam) and ab97240 goat polyclonal Secondary Antibody (Abcam). The relative concentration of the two chosen fractions was estimated by a densitometry quantification using ImageJ software [34]. The *in vitro* experiments have been performed using equal amount of purified hTop1 and hTop1Glu710Gly.

### DNA relaxation assays

The activity of 1 μl of hTop1 (16ng/μl) or hTop1Glu710Gly (16ng/μl) was assayed in 30 μl of reaction volume containing 0.5 μg of negatively supercoiled pBlue-Script KSII(+) DNA, that is present in both dimeric and monomeric forms and reaction buffer (20 mM Tris–HCl pH 7.5, 0.1 mM Na_2_EDTA, 10 mM MgCl_2_, 5 μg/ml acetylated bovine serum albumin and 150 mM KCl).

The effect of CPT on enzyme activity was measured by adding DMSO or 100μM of the drug to the reactions, that were stopped with 0.5% SDS after each time-course point at 37°C. The samples were resolved in a 1% (w/v) agarose gel in 48 mM Tris, 45.5 mM boric acid, 1 mM EDTA at 10 V/cm. The gels were stained with ethidium bromide (0.5 μg/ml), destained with water and photographed using a UV transilluminator.

### Cleavage kinetics

Oligonucleotide CL14 (5′-GAAAAAAGACTTAG-3′) that contain a hTop1 high affinity cleavage site, was 5′-end labelled with [γ^32^P] ATP. The CP25 complementary strand (5′-TAAAAATTTTTCTAAGTCTTTTTTC-3′) was 5′-end phosphorylated with unlabeled ATP. The two strands were annealed with a 2-fold molar excess of CP25 over CL14 [35]. The suicide cleavage reactions were carried out by incubating 20 nM of the duplex DNA with an excess of hTop1 or hTop1Glu710Gly enzymes at 25°C, in 20 mM Tris–HCl pH 7.5, 0.1 mM Na_2_EDTA, 10 mM MgCl_2_, 5 μg/ml acetylated BSA, and 150 mM KCl, in a final volume of 50 μl [36]. At various time points 5 μl aliquots were removed and the reaction stopped with 0.5% (w/v) SDS. After ethanol precipitation samples were resuspended in 5 μl of 1 mg/ml trypsin and incubated at 37°C for 60 minutes. However a short trypsin resistant peptide is always left explaining why the CL1 migrates slower than the CL14 oligonucleotide [20]. Samples have been analyzed by denaturing 7 M urea/20% polyacrylamide gel electrophoresis in TBE (48 mM Tris, 45.5 mM Boric Acid, 1 mM EDTA).

Oligonucleotide CL14-U (5′-GAAAAAAGACTUAG-3′) was 5′ end labelled and annealed with CP25 as for the CL14. 20 nM substrate has been incubated with an excess of hTop1 or hTop1Glu710Gly enzymes in 20 mM Tris–HCl pH 7.5, 0.1 mM Na_2_EDTA, 10 mM MgCl_2_, 5 μg/ml acetylated BSA, 150 mM KCl, at 25°C in a final volume of 40 μl. At various time points 5 μl aliquots were removed and the reaction stopped with 0.5% (w/v) SDS and directly loaded without ethanol precipitation and trypsin digestion. Samples have been analyzed by denaturing 7 M urea/20% polyacrylamide gel electrophoresis in TBE (48 mM Tris, 45.5 mM Boric Acid, 1 mM EDTA). In both experiments the percentage of cleaved substrate (CL1) was determined by PhosphorImager and ImageQuant software and normalized on the total amount of radioactivity in each lane.

### Religation kinetics

20 nM of CL14/CP25 (radiolabeled as previously described in the cleavage kinetic experiment) was incubated with an excess of hTop1 or hTop1Glu710Gly for 60 minutes at 25°C followed by 30 minutes at 37°C in 20 mM Tris–HCl pH 7.5, 0.1 mM Na_2_EDTA, 10 mM MgCl_2_, 50 μg/ml acetylated BSA, and 150 mM KCl. After the formation of the cleavage complex (CL1) a 5μl aliquote was removed and used as time 0 point, then DMSO or 100 μM CPT were added and religation reaction was started by adding a 200-fold molar excess of R11 oligonucleotide (5′-AGAAAAATTTT-3′) over the CL14/CP25 [20]. 5 μl aliquots were removed at various time points, and the reaction stopped with 0.5% SDS. After ethanol precipitation, samples were resuspended in 5 μl of 1 mg/ml trypsin and incubated at 37°C for 60 minutes. Samples were analyzed by denaturing 7 M urea/20% polyacrylamide gel electrophoresis in 48 mM Tris, 45.5 mM Boric Acid, 1 mM EDTA. The percentage of remaining cleavage complex was quantified by ImageQuant software, normalized to the total radioactivity for each lane and to the value at t=0 and finally plotted as a function of time.

### Molecular dynamics

The initial configuration of the wild type hTop1, in covalent complex with a 22 base pair linear double helix DNA substrate, has been modeled from the crystallographic structures (PDB 1K4S and 1TL8, respectively) as already reported [23].

The Glu710Gly mutant was generated using the rotamer module presents in the Chimera package [37]. The systems have been modeled using the AMBER03 all-atom force field [38] implemented by Sorin and Pande [39] in the GROMACS MD package version 4.5.4 [40]. The proteins was placed in a rhombic dodecahedron box with a minimum distance of 14 Å from the box edges, then filled with water molecules described by means of the TIP3P rigid potential and Na+ counter-ions [41] were added to neutralize DNA-enzyme total charge via the genion tool of the GROMACS package. The resulting WT system is composed of 9456 protein atoms, 1400 DNA atoms, 58919 water molecules, 20 Na+ ions, for a total of 187633 atoms.

Particle Mesh Ewald method (PME) was used to handle long-range electrostatic interactions using a cutoff of 1.2 nm in real space and the same cutoff value was used for Van der Waals interactions [42]. The LINCS algorithm was used to constrain bond lengths and angles [43]. Relaxation of solvent molecules and Na+ ions was initially performed keeping solute atoms restrained to their initial positions, in three cycles with decreasing force constant of 1000, 600 and 300 kJ/(mol N nm), for 300 ps each. The two systems have then been simulated for 75 ns with a time step of 2.0 fs and the neighbor list was updated every 10 steps. Temperature was kept constant at 300 K using the velocity rescale Berendsen method with a coupling constant of 0.1 ps during sampling, while pressure was kept constant at 1 bar using the Parrinello-Rahman barostat with a coupling constant of 1.0 ps during sampling [44].

Standard analyses, as root mean square deviations (RMSD) and fluctuations (RMSF), hydrogen bonds, distances evaluations etc. were calculated using tools of GROMACS MD 4.5.3 [40] package. Correlation maps were calculated on the C-alpha atoms as described [18] with in-house written codes. Images were produced with VMD [45] and Chimera [37].

## Abbreviations

hTop1: Human topoisomerase IB; CPT: Camptothecin; DMSO: Dimethyl sulfoxide; TPT: Topotecan; CPT-11: Irinotecan; PMSF: Phenylmethanesulfonylfluoride; MD: Molecular dynamics; RMSD: Root mean square deviation; RMSF: Root mean square fluctuation.

## Competing interests

The authors declare that they have no competing interests.

## Authors' contributions

CT, AO, BA and LZ carried out the experimental part of the manuscript. BMDR, AC and ID carried out the modeling and molecular dynamics simulations and analyses. PF and AD designed and coordinated the studies and CT, BMDR, PF and AD co-wrote the manuscript. All authors read and approved the manuscript.

## Supplementary Material

Additional file 1 **RMSD.** Root mean square deviation from the starting structure for wild type and Glu710Gly mutant. Plots were calculated for the core of the protein (black and green lines) and the whole protein including the linker (red and blue lines) for wild type and mutant respectively. Click here for file
