# Supplementary material for: Molecular mechanism of the camptothecin resistance of Glu710Gly topoisomerase IB mutant analyzed in vitro and in silico
Source: Mol Cancer. 2013 Sep 3;12:100. doi: 10.1186/1476-4598-12-100 (PMC3766703; doi:10.1186/1476-4598-12-100)

Additional file 1. RMSD. Root mean square deviation from the starting structure for wild type and Glu710Gly mutant. Plots were calculated for the core of the protein (wild type in black and mutant in green lines) and the whole protein including the linker (wild type in red and mutant in blue lines).

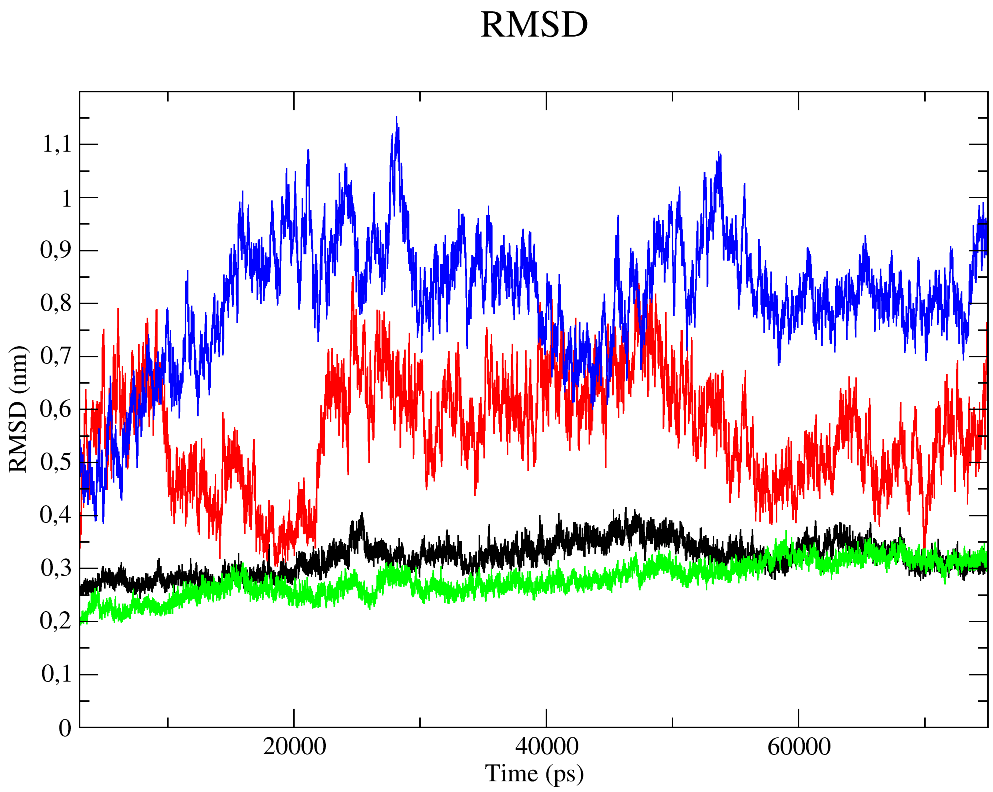

Supplement: Additional file 1 — RMSD. Root mean square deviation from the starting structure for wild type and Glu710Gly mutant. Plots were calculated for the core of the protein (black and green lines) and the whole protein including the linker (red and blue lines) for wild type and mutant respectively. [file 1476-4598-12-100-S1.pdf]
